# Tracing the Scientific Legacy: Bibliometric Analysis of LATAM Research in Bariatric Surgery for 33 Years

**DOI:** 10.1007/s11695-024-07339-6

**Published:** 2024-07-13

**Authors:** Gonzalo Andrés Domínguez Alvarado, Luis Ernesto López Gómez, Gustavo Adolfo Serrano Baez, Sergio Eduardo Serrano Gómez, Andrés Vásquez Pineda, Tatiana Bustos Lopez, María Alejandra Arévalo González, Carlos Felipe Palomino Peña, Luis David Chavarría Granda, Daniela Álvarez Leon, Diego Mauricio Barrera Arguello

**Affiliations:** 1 Department of Surgery, Innovation and Research in Surgery, Universidad Autónoma de Bucaramanga, Bucaramanga, Santander, Colombia; 2 Department of Surgery, Universidad Autónoma de Bucaramanga, Bucaramanga, Santander, Colombia; 3 Adult Critical and Intensive Care Unit, Universidad Autónoma de Bucaramanga, Bucaramanga, Santander, Colombia; 4 Faculty of Health Sciences, Universidad Autónoma de Bucaramanga, Bucaramanga, Santander, Colombia; 5 Department of Surgery, Innovation and Research in Surgery, Autonomous University of Bucaramanga, Bucaramanga, Santander, Colombia; 6 Hospital San Vicente Fundación Rionegro, Rionegro, Antioquia Colombia; 7 Universidad de Santander, Bucaramanga, Santander, Colombia

**Keywords:** Bariatric medicine, Latin America, Obesity

## Abstract

**Introduction:**

Metabolic and bariatric surgery (MBS) has experienced considerable growth, addressing the challenges of obesity and its complications. The lack of a comprehensive bibliometric analysis in Latin America motivates this study, highlighting the need to understand the evolution of research in this area and its impact on clinical decision-making and health policies.

**Methodology:**

A cross-sectional bibliometric study was carried out using the Scopus database. A structured search strategy was designed to identify articles related to bariatric surgery with authors affiliated with Latin American countries. Inclusion and exclusion criteria were applied, followed by a descriptive and bibliometric analysis of the scientific production found.

**Results:**

A total of 3553 documents published between 1991 and 2024 were included. There was an annual growth of 11%, with an average age of documents of 7.5 years. A concentration was observed in some countries, notably Brazil, Mexico, and Chile. Although scientific output increased, the average number of citations per article showed a downward trend since 2003.

**Discussion:**

Despite the growth in scientific production, the quality and relevance of research is questioned, especially given the decrease in the impact received. It highlights the lack of meaningful regional collaboration, which could limit the sharing of knowledge and resources. Questions are raised about gaps in research capacity and the economic and development implications are discussed.

**Conclusions:**

This study provides valuable information to strengthen future research in bariatric surgery in Latin America. It highlights the importance of promoting regional and international collaboration and improving research training in countries with less participation. Clinical intervention strategies can benefit from better understanding research trends and adopting evidence-based practices in a more informed manner.

**Graphical Abstract:**

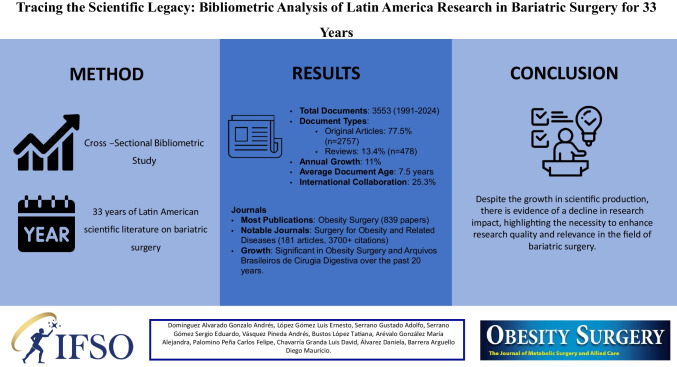

## Introduction

Metabolic and bariatric surgery (MBS), as a sub-specialized branch of general surgery, has undergone considerable expansion in recent decades, effectively addressing the challenges of obesity and its associated consequences [1, 2]. This crucial field of health has received increasing attention from the scientific community, driving a proliferation of research and scholarly contributions that have enriched our understanding of clinical practices, treatment trends, and long-term outcomes [3].

Bibliometrics makes it possible to perform a numerical analysis of publications within a specific field and time period, as well as to examine the relationships between these publications. On the other hand, citation analysis is a bibliometric method that is based on the identification of citations in scientific documents and on the relationships between authors or articles, represented through a website or graph [4]. Likewise, advanced bibliometric analysis is used as a robust approach to study the popularity of publications, authors, institutions, and countries, in addition to the international effects of scientific research and the flow of interdisciplinary information [4].

Faced with this dynamic and constantly evolving scenario, there is an imperative need to carry out an exhaustive bibliometric analysis of Latin America research in MBS. The importance of this study lies in several key factors.

First, Latin America has emerged as a relevant player in global medical research, standing out for its diversity, cultural richness, and significant impact on various disciplines (plastic surgery, pediatric, general surgery, internal medicine, and bariatric surgery) [5–8]. However, MBS, despite its importance for public health, has not been the subject of a comprehensive bibliometric analysis in the region.

Second, obesity and its associated complications represent a growing public health challenge in Latin America. According to 2016 data, adult obesity affected 24.2% of the adult population in the region, equivalent to 106 million adults, considerably higher than the global average of 13.1% [9–11]. Given this alarming situation, the demand for surgical interventions to effectively address this problem has increased significantly. Therefore, bibliometric analysis of research in MBS will not only contribute to understanding the evolution of knowledge in this area but will also provide a solid basis for clinical decision-making, health policy, and future research.

Third, international collaboration has been a key driver of scientific advancement, and understanding how Latin America integrates into this global network is essential to fostering collaboration and synergy [12, 13]. This study will identify the connections and relationships that exist between researchers and institutions in the region with their international counterparts, thus highlighting the position of MBS research in the global context [9, 14].

Finally, this bibliometric analysis aims to fill a gap in the scientific literature by offering a comprehensive and detailed view of bariatric surgery research in Latin America over 33 years. By addressing these justifications, we aspire to contribute to the continued growth and development of bariatric surgery research, providing valuable perspectives that will benefit the scientific community, healthcare professionals, and ultimately patients undergoing those procedures.

## Material and Methods

### Study Design

This is a cross-sectional bibliometric study.

### Databases

The largest database of peer-reviewed scientific literature, Scopus, was used as a data source for this analysis. Previously, the reasons for the use of this base have been described [10–12]. In addition, unlike other search engines, citation indexes, and databases, such as PubMed or Web of Science, Scopus has the highest number of indexing Latin American biomedical journals.

### Search Strategy

A structured search was designed and executed to identify articles related to any research approach in bariatric surgery whose authors had a Latin affiliation. For this, the affiliation reported in the metadata was considered and corroborated by the official full-text publication. The search strategy was built using MeSH terms, as well as synonyms, in both English and Spanish. After a pilot test, we defined using the following search: [13, 15] TITLE-ABS-KEY(“bariatric surger*”) OR TITLE-ABS-KEY(“Metabolic surger*”) OR TITLE-ABS-KEY(“Weight loss surgery”) OR TITLE-ABS-KEY(“diabetes surgery”) OR TITLE-ABS-KEY(“Gastric bypass”) OR TITLE-ABS-KEY(“gastric band*”) OR TITLE-ABS-KEY(“sleeve gastrectomy”) AND AFFILCOUNTRY (Antigua AND Barbuda) OR AFFILCOUNTRY (Argentina) OR AFFILCOUNTRY (Bahamas) OR AFFILCOUNTRY (Barbados) OR AFFILCOUNTRY (Belize) OR AFFILCOUNTRY (Bolivia) OR AFFILCOUNTRY (Brazil) OR AFFILCOUNTRY (Chile) OR AFFILCOUNTRY (Colombia) OR AFFILCOUNTRY (costa AND Rica) OR AFFILCOUNTRY (Cuba) OR AFFILCOUNTRY (Dominican) OR AFFILCOUNTRY (Ecuador) OR AFFILCOUNTRY (el AND Salvador) OR AFFILCOUNTRY (Grenada) OR AFFILCOUNTRY (Guatemala) OR AFFILCOUNTRY (Guyana) OR AFFILCOUNTRY (Haiti) OR AFFILCOUNTRY (Honduras) OR AFFILCOUNTRY (Jamaica) OR AFFILCOUNTRY (Mexico) OR AFFILCOUNTRY (nicaragua) OR AFFILCOUNTRY (Panama) OR AFFILCOUNTRY (Paraguay) OR AFFILCOUNTRY (Peru) OR AFFILCOUNTRY (Dominican AND republic) OR AFFILCOUNTRY (saint AND lucia) OR AFFILCOUNTRY (suriname) OR AFFILCOUNTRY (trinidad AND tobago) OR AFFILCOUNTRY (uruguay) OR AFFILCOUNTRY (venezuela).

### Standardization and Data Collection

Considering that in Latin America the predominant languages are Spanish and Portuguese, documents were included in English, Spanish, and Portuguese languages. This search, which was conducted until February 2, 2024, was filtered with the tag “Journals.” Thus, literature that does not follow the regular peer review process for publication in scientific journals, such as books, book series, abstracts, and reports of scientific events, was excluded. No time limit was established for the inclusion of evidence.

After this first phase, a manual review was carried out to eliminate duplicates and those articles not related to the topic of interest, based on title, abstract, and keywords, in Microsoft Office Excel 2016. Finally, another manual review was carried out, in order to standardize the data of the variables of interest and reduce the discrepancies between the way in which the metadata was originally recorded. In this way, categories were regrouped. For example, in the case of article typology, all original articles, regardless of observational or experimental design, were categorized as “Articles.” Reviews, regardless of their design (whether narrative, systematic, or meta-analysis), were categorized as “Reviews.” Case series and case reports were categorized as “Case Reports,” and editorials, letters to the editor, comments, etc.; in the same way, to ensure homogeneity and specificity in Latin America affiliations, these were reviewed and corroborated [13, 15].

### Statistical, Visual, and Bibliometric Analysis

Network and bibliometric metrics were used to establish trends, characteristics, and scientific impact. All documents that met the inclusion criteria were included in the overall analysis. The R bibliometric package was used to perform this analysis, which allows the calculation of quantitative bibliometric indicators, as well as the visualization of the results (version 4.3.1). Synonyms, errors, plurals, and variants were regrouped. In this way, keywords and institutions were standardized [16].

A descriptive analysis of the scientific production was carried out, and impact indicators were calculated. To measure the impact of institutions and countries, the h-index, g-index, and m-index metrics were used, as well as the absolute value of cumulative citations. The definitions and specifications of the use of these metrics in bibliometric studies have been previously described. Lotka’s law was used. This law describes the frequency of publication by authors in any given field. The calculation of frequencies and percentages was carried out using Microsoft Office Excel 2016. [17].

### Ethical Statements

This study did not require approval by an ethics committee, considering that it did not conduct research on human subjects, biological models, or medical history.

## Results

**Fig. 1:**
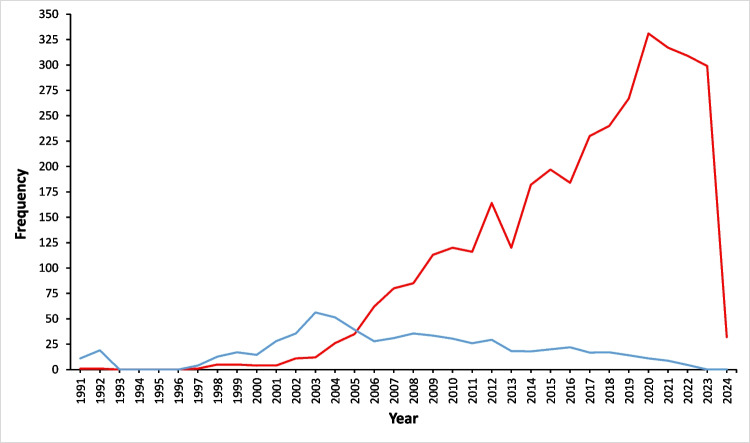
Annual scientific growth of Latin America bariatric surgery research since 1991. Red: annual publication frequency. Blue color: average number of citations received per article per year

A total of 3553 documents were included, after the application of inclusion and exclusion criteria, published between 1991 and 2024, constituting an evaluation time window of 33 years. 77.5% (*n* = 2757) of the documents were original articles, followed by 13.4% (*n* = 478) of revisions. An annual growth of 11%, an average age of documents of 7.5 years, and an international collaboration of 25.3% were identified (Table 1). A sustained growth in scientific production was visualized from 2001 onwards, with a peak in 2020 and then a decline until 2023. Different from the impact received, it was determined by the average number of citations per article per year, which has been fluctuating with a downward trend since 2003 (Fig. 1). When applying Lotka’s law, it was observed that 75.4% of the identified authors have only published a single document, followed by 13.7% with two documents.

**Table 1:** General characteristics of Latin American scientific production on bariatric surgery (*N* = 3553)

	*n*	%
Type of article		
Original article	2757	77.5
Revision	478	13.4
Case report	3	0.1
Letters*	315	9
Authors		
Authorship	13.961	-
Authors of documents with sole authorship (*N* = 13,961)	112	0.80
Collaboration		
Articles with sole authorship	152	-
Co-authorship per article (average)	6.7	-
International co-authorship	25.3	-
Keywords	11.419	-
Journals	772	-
Average age of documents (years)	7.5	-
Average citations per document	16.8	-
Annual growth	-	11.07

^*^Includes letters to the editor, editorials, and comments

By characterizing the production of institutions, and countries, it was revealed that of the most prolific institutions, four are Brazilian and the remaining are Chilean. The University of São Paulo (Brazil) is the institution with the highest number of documents published in the region (*n* = 593), as well as the impact obtained (h-index of 48). In terms of countries, Brazil leads the production and impact (2322 articles published and h-index of 83), followed by Mexico and Chile. Even so, Chile is the Latin American country that, after Brazil, has the second highest impact (h-index of 62) (Table 2).

**Table 2:** Characterization of affiliations, and most prolific Latin American countries on bariatric surgery research

Affiliation	Documents over time				Total bariatric surgery documents	Index h	Country
	1991–2000	2001–2010	2011–2020	2021–2024			
University of Sao Paulo	2	104	331	156	593	48	Brazil
State University of Campinas	0	25	116	45	186	32	Brazil
Federal University of São Paulo	0	20	111	44	175	27	Brazil
Federal University of Pernambuco	0	15	91	29	135	22	Brazil
University of Chile Clinical Hospital	2	51	64	5	122	34	Chile
Country	Documents over time				Total bariatric surgery documents*	Index h	
	1991–2000	2001–2010	2011–2020	2021–2024			
Brazil	4	354	1353	611	2322	83	
Mexico	13	55	206	169	443	40	
Chile	2	103	237	66	408	62	
Argentina	1	15	119	68	203	29	
Colombia	0	13	73	54	140	17	

^*^Production was counted individually. Therefore, a document could have been counted several times based on international collaboration

**Fig. 2:**
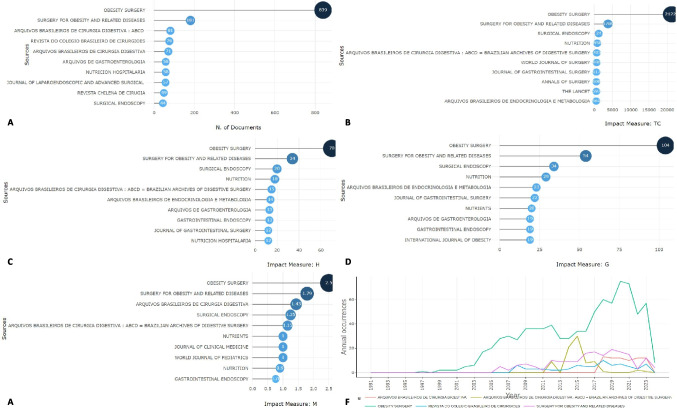
Impact obtained and frequency of publication in journals with the highest number of Latin America articles on bariatric surgery research. **A** Frequency of articles published. **B** Total number of citations received. **C** H-index of articles. **D** Index g of articles. **E** Index m of articles. **F** Annual frequency of publication of Latin America articles on bariatric surgery in the five most popular journals

*Obesity Surgery* is the journal with the highest number of papers published in Latin America research on bariatric surgery (839 papers) (Fig. 2A), as well as impact obtained, in all metrics (Fig. 2B–E). It is followed by *Surgery for Obesity and Related Diseases* with 181 published articles and more than 3700 citations. When visualizing the evolution of journals over time, *Obesity Surgery* and *Arquivos Brasileiros de Cirurgia Digestiva* have had the most notable growth in the last 20 years (Fig. 2F).

**Fig. 3:**
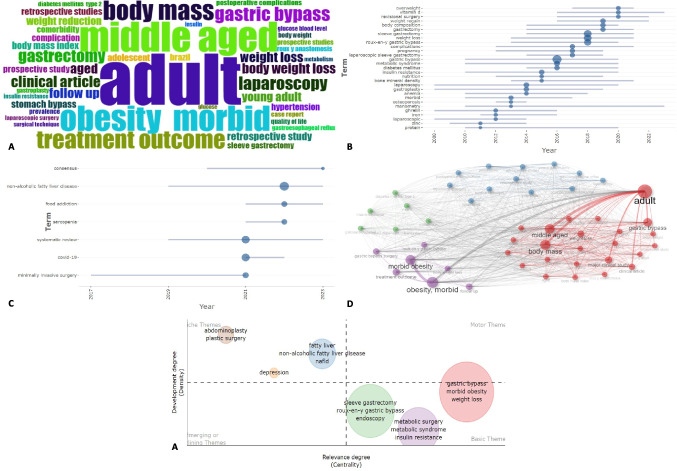
Evolution and trends of Latin America research in bariatric surgery. **A** Most frequent keywords cloud in. **B** Frequency of topics between 2010 and 2020. **C** Frequency of topics as of 2021. **D** Keyword co-occurrence network. **E** Thematic map on degree of relevance and development of topics

Regarding the historical patterns and trends of Latin America research in bariatric surgery, based on the frequency of occurrence of keywords, it was revealed that clinical research, essentially retrospective, focused on the evaluation of treatment outcomes in adults, has been the most frequently studied in the region (Fig. 3A). Compared to previous decades, in the 2010s there was a particular interest in the study of micronutrients, osteoporosis, anemia, and insulin resistance, related to surgical techniques such as sleeve gastrectomy and Y-de-Roux gastric bypass (Fig. 3B). As of 2021, there has been a special emphasis on data systematization, sarcopenia, non-alcoholic fatty liver disease, COVID-19, and consensus building (Fig. 3C). By constructing a co-occurrence network, different from the one previously mentioned, it is also possible to observe research niches related to the metabolic outcomes of remission of diabetes mellitus, assessment of outcomes linked to gastroesophageal reflux and postoperative complications (Fig. 3D). Finally, the visualization of the thematic map with respect to the degree of relevance and development of subtopics showed that, unlike the already known base topics, the outcomes of depression, fatty liver, and aesthetics are emerging lines (Fig. 3E).

**Fig. 4:**
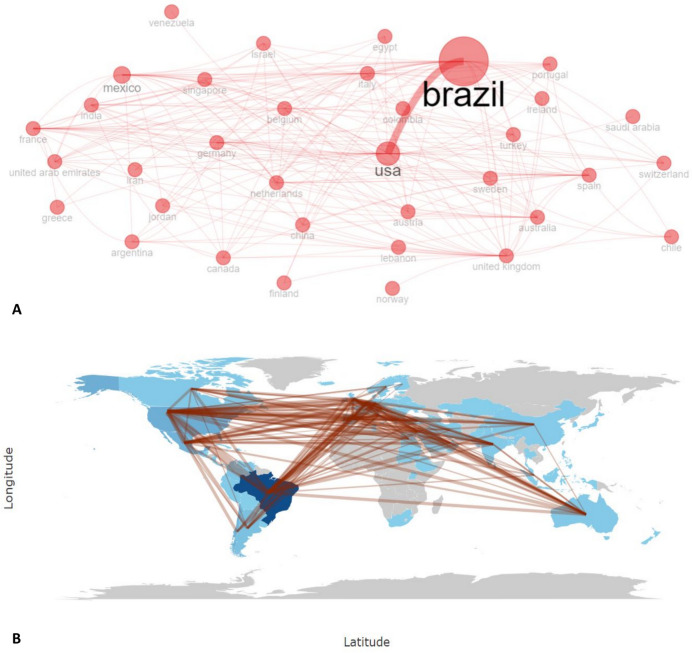
Collaboration networks of countries in Latin American research in bariatric surgery. **A** Strength of collaboration between countries. **B** Frequency of collaboration between countries

In terms of regional and intercontinental collaboration, it was demonstrated that Brazil has a solid network, mainly with the USA and some European countries, but also with Latin American collaboration. Mexico is the country that follows in strength and frequency of collaboration, with a broad network of collaboration with Asian, European, and Latin American countries (Fig. 4A). Argentina and Chile are also countries in the region that tend to collaborate frequently with the USA, Europe, and Asia (Fig. 4B).

As for the studies that have historically obtained the greatest impact to date, they are as follows: [1] Bariatric Surgery and Endoluminal Procedures: IFSO Worldwide Survey 2014 (521 citations, published in *Obesity Surgery*, 2018, with the participation of Colombia); [2] Long-term Weight Regain after Gastric Bypass: A 5-year Prospective Study (490 citations, published in *Obesity Surgery*, 2008, with participation from Brazil); and [3] Bariatric Surgery Worldwide: Baseline Demographic Description and One-Year Outcomes from the Fourth IFSO Global Registry Report 2018 (473 citations, published in *Obesity Surgery*, 2019, with the participation of Brazil).

## Discussion

Over the last 3 decades, cases of obesity have increased dramatically, from approximately 7% to 25% [18]. In addition, it is estimated that currently more than two billion people around the world are overweight or obese; these data reveal the severity of this disease as a public health problem, which justifies the need for high-quality research and scientific production in order to discover and innovate solutions to address this problem [19].

This bibliometric analysis provides an idea of the status and trend of bariatric surgery research in Latin America during the last 3 decades. Although there has been a sustained growth in scientific production, especially since 2001, it is important to question the quality and real impact of this research to have a clear and critical understanding of the current panorama. Despite the increase in the number of publications, the average number of citations received per article has been on a downward trend since 2003, suggesting that a considerable portion of the research may lack relevance or may not be addressing the most pressing needs in this field.

On the other hand, when examining the distribution of contributions, a concentration is shown in a few countries and research centers in the region. Brazil stands out as a leader in scientific production and impact in bariatric surgery, closely followed by Mexico and Chile, both Latin American countries with relevant research contributions. This disparity with respect to other Latin American nations raises questions about gaps in research capacity, available resources, and priorities set in different countries. Bariatric surgery research may not be receiving adequate attention in some nations, despite the increasing prevalence of obesity and its associated complications across the region.

Another striking aspect is the apparent lack of meaningful regional collaboration. Although there are international collaboration networks, with countries in North America, Europe, and Asia, cooperation among Latin American countries themselves seems limited. This fragmentation can be an obstacle to the exchange of knowledge, the optimization of resources, and the joint approach to shared challenges in the region. It is essential to foster greater synergy and take advantage of the complementary strengths of bariatric surgery research groups in Latin America.

A robust and extensive bibliometric study recently published by the International Federation for Bariatric Surgery (IFSO) yields important data for Latin America and the world. This study by Corrêa et al. reveals the state of the scientific literature on bariatric surgery over the past 71 years. The authors observed that scientific production is overwhelmingly led by the USA, with 65.7% of publications. However, the analysis by restricted region shows that Brazil is the most productive country in Latin America, accounting for 7.5% of global production. This suggests that, although the USA leads research globally, Brazil could assume a leading role in the progress of bariatric surgery in Latin America [18].

The authors describe that the growth of scientific publications has been exponential since 2000, figures that are consistent with our findings. This increase, according to Corrêa et al., is related to the increase in the frequency of obesity and the subsequent need for surgeries to address both obesity and its associated diseases [18].

In addition, other global bibliometric analyses have also revealed findings similar to those obtained in our study. In their paper entitled “The Evolution of Bariatric Surgery Publications and Global Productivity: A Bibliometric Analysis” [20], Zeki Ozsoy and Emre Demir examined scientific publications from 1980 to 2016, finding extremely interesting data. Regarding Latin America, the authors concluded that Brazil is one of the countries with the highest number of publications and growth in research on bariatric surgery, above France, Spain, and Germany, and the authors observed, as we do, that scientific production has been increasing since 2001. However, Ozsoy and Demir [20] described that the number of citations shows an increasing trend at the global level, unlike our analysis that shows a decrease for Latin America; therefore, the question arises as to why this behavior in Latin America [20].

Ozsoy and Demir explore an economic and development aspect that could shed light on the disparity in research capacity in Latin America. These authors recognize the impact that variables such as gross domestic product (GDP), GDP per capita, and the Human Development Index (HDI) have on scientific production in the field of bariatric surgery. The authors take as an example the USA, a country with much more favorable economic variables than most Latin American countries. According to Ozsoy and Demir, this is reflected in the considerable budget allocated for research, the abundance of research centers, and the growth in the prevalence of obesity in the USA. These variables tend to be limited in some Latin American countries [20].

In addition to this, studies such as those of Debi et al. [21] and Corrêa et al. [22] support the relationship between the amount of scientific production in bariatric surgery and the country’s GDP, in addition to total health expenditure and the prevalence of obesity. Likewise, the study by Paolino et al. [18, 22] observes a relationship between higher scientific production in higher-income countries compared to low-income countries, so it can be concluded that economic and development indicators have a significant impact on a country’s scientific production.

Although limitations have been identified, this study offers valuable information that can be used to strengthen and guide future research in bariatric surgery in the region. By revealing the strengths and weaknesses present, it is possible to create concrete strategies to address the deficiencies that exist. For example, these findings can be used to inform how to allocate resources and set research priorities in countries and centers with lower scientific output. Likewise, by recognizing emerging subject areas and promising research niches, researchers can more effectively coordinate their efforts with present and future needs.

Our work highlights the importance of promoting greater regional and international collaboration in bariatric surgery research from a health policy perspective. This information can be used by governments and health entities to plan programs that encourage the formation of research networks, the sharing of knowledge, and the efficient use of resources. Also, the findings can support the establishment of specific training and education programs to improve research skills in countries with lower participation. Clinical intervention strategies can benefit from a deeper understanding of research trends, which would facilitate the more informed adoption of evidence-based practices and a more personalized approach in diverse local contexts.

## Conclusions

Despite the growth in scientific production, there is evidence of a decrease in the impact of this research, which suggests the need to improve the quality and relevance of research in the field. The concentration of scientific output in a few countries highlights the importance of addressing gaps in research capacity and available resources in different regions. In addition, the lack of meaningful regional collaboration poses challenges for the exchange of knowledge and resources among Latin American countries. These findings underscore the importance of promoting regional and international collaboration, as well as strengthening research training in countries with lower participation. By better understanding research trends and adopting evidence-based practices in a more informed manner, more effective clinical intervention strategies can be designed to address obesity and its associated complications in the region. Ultimately, this study provides a solid foundation to guide future research in bariatric surgery in Latin America, with the goal of improving the health and well-being of the population.
